# Altered Gene Synchrony Suggests a Combined Hormone-Mediated Dysregulated State in Major Depression

**DOI:** 10.1371/journal.pone.0009970

**Published:** 2010-04-01

**Authors:** Chris Gaiteri, Jean-Philippe Guilloux, David A. Lewis, Etienne Sibille

**Affiliations:** 1 Department of Psychiatry, University of Pittsburgh, Pittsburgh, Pennsylvania, United States of America; 2 Center for Neuroscience, University of Pittsburgh, Pittsburgh, Pennsylvania, United States of America; 3 Faculté de Pharmacie, Université Paris-Sud EA 3544, Châtenay-Malabry, France; King Abdullah University of Science and Technology, Saudi Arabia

## Abstract

Coordinated gene transcript levels across tissues (denoted “gene synchrony”) reflect converging influences of genetic, biochemical and environmental factors; hence they are informative of the biological state of an individual. So could brain gene synchrony also integrate the multiple factors engaged in neuropsychiatric disorders and reveal underlying pathologies? Using bootstrapped Pearson correlation for transcript levels for the same genes across distinct brain areas, we report robust gene transcript synchrony between the amygdala and cingulate cortex in the human postmortem brain of normal control subjects (n = 14; Control/Permutated data, p<0.000001). Coordinated expression was confirmed across distinct prefrontal cortex areas in a separate cohort (n = 19 subjects) and affected different gene sets, potentially reflecting regional network- and function-dependent transcriptional programs. Genewise regional transcript coordination was independent of age-related changes and array technical parameters. Robust shifts in amygdala-cingulate gene synchrony were observed in subjects with major depressive disorder (MDD, denoted here “depression”) (n = 14; MDD/Permutated data, p<0.000001), significantly affecting between 100 and 250 individual genes (10–30% false discovery rate). Biological networks and signal transduction pathways corresponding to the identified gene set suggested putative dysregulated functions for several hormone-type factors previously implicated in depression (insulin, interleukin-1, thyroid hormone, estradiol and glucocorticoids; p<0.01 for association with depression-related networks). In summary, we showed that coordinated gene expression across brain areas may represent a novel molecular probe for brain structure/function that is sensitive to disease condition, suggesting the presence of a distinct and integrated hormone-mediated corticolimbic homeostatic, although maladaptive and pathological, state in major depression.

## Introduction

Major depression affects more individuals than all other psychiatric illnesses combined, and a significant number of patients do not remit after pharmacological or behavioral treatment [1], hence inflicting a continuous toll on affected individuals and on society [2]. Changes in the coordinated function of a neural network comprising cortical and subcortical brain areas are thought to underlie the mood regulation deficit in depression [3]. The functional connectivity between two critical components of this corticolimbic circuitry, the amygdala and anterior cingulate cortex, potentially mediates the relay of emotion-related information for cortical processing, and feedback regulation on amygdala activity [4]. In control non-depressed subjects, the volume, function and connectivity of these two areas are affected by serotonin-related gene variants [4], [5], together suggesting that this pathway may be recruited in diseases of altered mood. Indeed, recent findings suggest an increased task-related recruitment of rostral cingulate and decreased coactivation of amygdala and cingulate in depressed patients [6]. Moreover, studies in depression suggests functional, cellular and molecular pathologies in both areas [7]–[10]. So, alterations in the intrinsic circuitry of the amygdala and anterior cingulate cortex may result in altered connectivity, deficient cingulate feedback regulation on amygdala function and abnormal processing of emotion-related stimuli in depression [11], [12].

How can brain region activities be investigated at the molecular and gene levels? We hypothesized that pathological mechanisms leading to depression may affect the coordination of gene expression patterns in the brain, and tested this hypothesis within a set of related brain areas, the amygdala and the subgenual anterior cingulate cortex. Our assay makes use of the fact that correlations in gene transcript levels across samples and datasets (“coexpression” or “coregulation”) represent intrinsic attributes of cellular and biological systems [13], [14], including in the human brain [15], [16]. As expected for inter-related biological systems, many genes show variability in expression that do not reflect measurement error and that are consistently identified in a range of tissues and organisms [13], [14], [17]. These correlated relationships are *(i)* driven by various molecular mechanisms, genetic make-up and function-dependent synchronization, *(ii)* central to cellular function, *(iii)* link genes of common biological functions [13], [17], and thus can be used to create gene interactions networks [18]. Hence, based on indications that correlated expression profiles might serve as markers of cellular or tissue relationships, we investigated synchronized expression across two regions implicated in the altered mood component of major depression. Confirming our hypotheses, we show that gene-wise coordinated transcript levels is a robust component of expression across regions within subjects, and that major depression is associated with significant gene-specific alterations in amygdala-cingulate gene coordination.

## Results

### Large-scale gene transcript synchrony across brain areas

**Figure 1:**
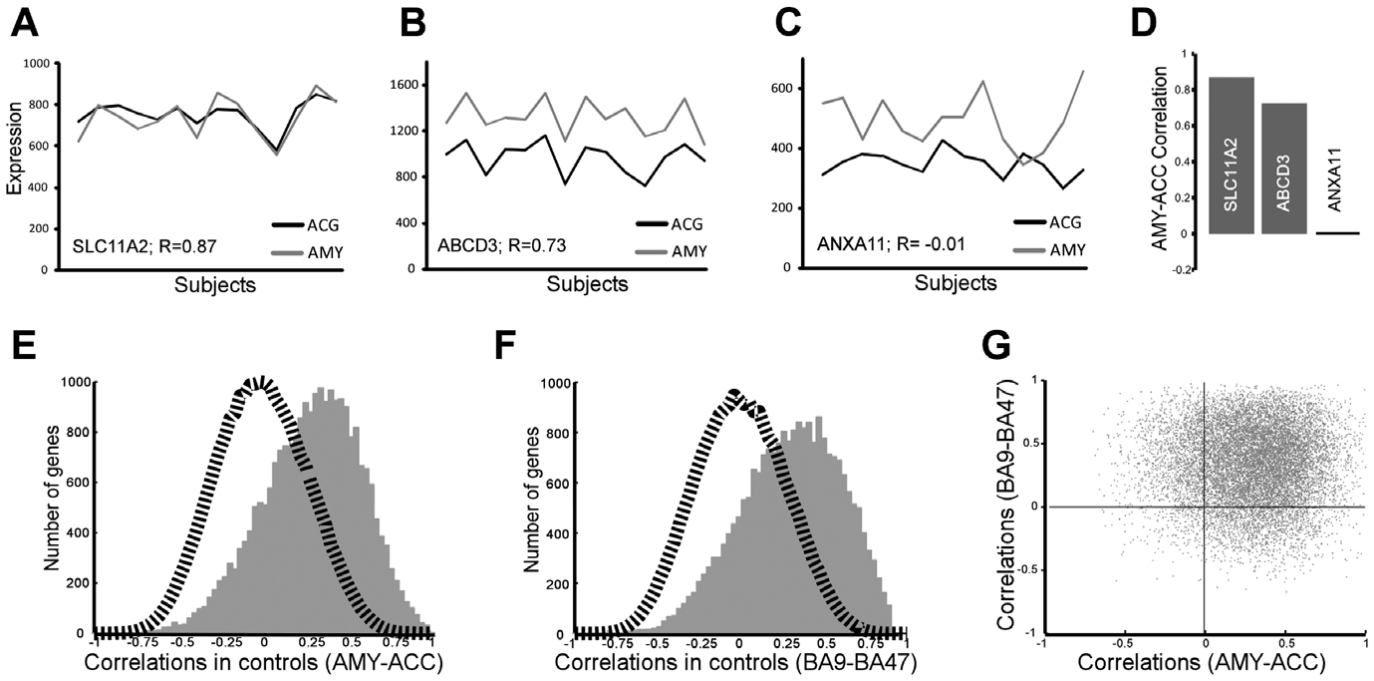
Correlated genewise transcript levels across brain regions. **A–D** Examples of within-subject positive, negative and absent amygdala-cingulate gene synchrony. **E** The right-shifted histogram of genewise transcript correlation suggests that the majority of genes are similarly regulated in both areas. The permutated data (dashed line) is centered on zero, indicating that gene coordination is subject-specific. **F** A similar pattern of gene synchrony was observed between two areas of the prefrontal cortex in an independent cohort (“BA”, Brodman area). **G** The lack of correlations in the extent of gene synchrony between the amygdala-cingulate and prefrontal cortical areas demonstrate that different sets of genes present coordinated transcript levels in the two different sets of brain areas (**G**, R = 0.002). AMY, amygdala, ACC, anterior cingulate cortex.

Transcripts for a particular gene are synchronized between two regions if they display significantly higher correlation across brain regions compared to permutated data. Here, using gene array data in the human postmortem brain of control subjects [10] (n = 14), we demonstrate that a large number of genes displayed positive correlations of transcript levels between amygdala and cingulate (Figure 1A–D), resulting in a unimodal distribution (Median r = 0.32) (Figure 1E). In contrast, the distribution of the permutated data, in which the subject linkage across regions was scrambled centered on the null correlation (Dashed line in Figure 1E; r = 0.014; Control/Permutated, p<0.000001). As no similar dataset are currently available (See Discussion), we investigated the presence of regional gene synchrony across a different set of brain areas. Large-scale gene synchrony was confirmed between two pre-frontal cortical regions in an independent cohort [19] (n = 19 subjects), and affected different genes (Figure 1F–G).

### Gene coordination is not a result of age-related changes in gene transcript levels

**Figure 2:**
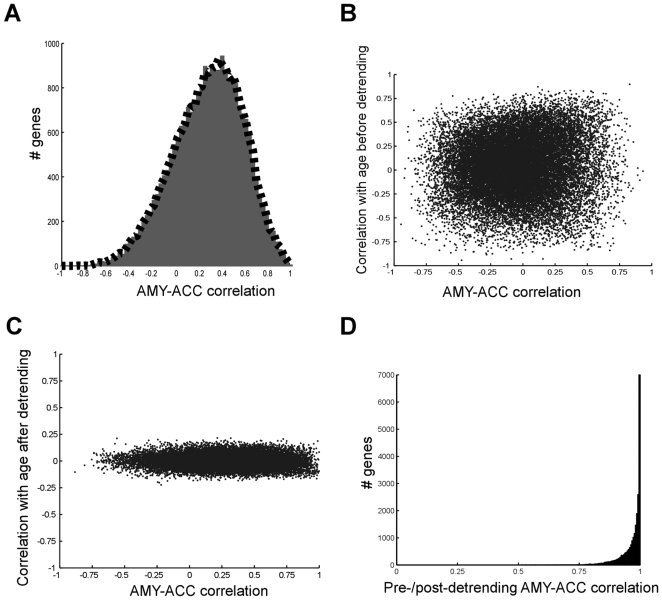
Age-related genes do not significantly influence gene coordination. **A** Age-detrended bootstrapped estimates of gene coordination were significantly different from the null permutated model (p<.00001; distribution outlined by black dashed line) and did not decrease the overall levels of gene coordination, when compared to the original bootstrap estimates (red distribution). **B–C** Relationships between amygdala-cingulate coordination and age correlation, before (**B**) and after (**C**) removal of any age-correlation indicated that age-detrending did not affect amygdala-cingulate regional coordination. As shown in **C**, the distribution of age correlations is centered on zero and highly compressed compared to figure **B**. The residual spread in the y-axis is due to inevitable, small, randomly occurring correlations while resampling the detrended data. **D** Histogram of correlations between original and detrended amygdala-cingulate coordination levels, showing that the vast majority of genes retain highly similar regional correlation, thus demonstrating an overall very small contribution of age to regional gene synchrony.

Age-related genes could produce correlations between gene expression levels across samples because numerous genes have strong correlations with age [19] and our subjects are acquired from a range of ages. It is therefore possible that the permutation destroys the global shift towards higher correlation (Figure 1E) simply because it scrambles the age-induced correlations. To investigate this putative effect, we subtracted age as a contributing factor and recomputed regional gene transcript correlations (see Methods and Figure 2B–D for details on age-detrending). As figure 2A shows, the entire shift towards positive correlation values across regions via gene coordination was achieved without any age-correlations in the expression profiles, demonstrating that the gene expression correlations with age have only an exceedingly small influence on regional gene coordination.

A genetic confound for these effects was also extremely unlikely as allelic frequencies for very large gene numbers would need to be similar within cohort, while being significantly different across cohorts, in order to generate the observed differences. Moreover, transcripts with reported impact of genetic variant on array hybridization and signal level [20] displayed low synchrony in both sets of brain regions (i.e. COMT; r≤0.2, p>0.05), thus ruling out technical confounders. Finally, regional gene coordination was observed across a heterogeneous cohort and could not distinguish the contribution of individual demographic or clinical factors (e.g. male/female difference, drug exposure; File S1).

### Gene transcript coordination is not a result of microarray gene expression protocols

RMA-based methods (as used here) have superior low-level detection capability compared to GCOS-MAS5 [21]; however, to ensure that normalization method was not responsible for the observed correlations, we recomputed the cross-area correlations using MAS5-normalized data. We observed similar and highly significant shifts (p<.000001) to positive levels of coordination for both amygdala-cingulate and prefrontal cortex regions using MAS5-normalized data, as we did with GCRMA-normalized data (not shown). Moreover, normalization-based differences in the estimated level of coordination were consistent low for highly coordinated genes, which are the focus of this study.

### Technical reliability of array data by quantitative real-time PCR (qPCR)

qPCR confirmation typically relies on measuring group differences 20% or greater in magnitude and averaged over multiple samples. Accordingly, the technical reliability of the array data used here was previously validated by independent qPCR measurements (array/qPCR correlation r≥0.75; n = ∼20 genes) [10]. While independent verification of differential expression changes between groups is a preliminary condition to establish confidence level in the quality of the gene array dataset before pursuing gene coregulation studies, measures of coordinated gene expression rely on changes of small magnitude across individual samples (Main text; Figure 1A–C) that are typically within the margin of technical variability in qPCR. For instance, we performed qPCR on 18 additional genes with coordinated regional expression (ATP5G1, CRHBP, MAOB, NFE2L2, PHKB, POLR2E, PRKAG2, RXRA, SAT1, AACS, CAP1, CDC42, CRYZ, GRLF1, IRF2BP2, NEFL, PAPOLA and SCN2A2). Samples were run in quadruplicates based on three internal controls (ACT, GAPDH and CYC). Transcript changes associated with variable coregulation levels resulted into lower coefficients of variation by array quantification (CV_array_ = 0.19 in amygdala and cingulate) compared to qPCR values (CV*_qPCR_* = 0.37; p<0.005 in amygdala; p<0.0001 in cingulate, two-group *t-*tests). This higher CV*_qPCR_* potentially reflects the exponential amplification of PCR reactions compared to the linear hybridization detection by arrays, and effectively limits the potential of confirming coregulation by qPCR in this and related studies. These results are consistent with other studies on microarray-based gene network, where qPCR is typically used to validate the mean absolute expression level [22], [23]. Instead, the validity and biological relevance of coordinated gene expression typically relies on extensive alternative forms of conformation through combining datasets and through permutation testing procedures (both performed here) [13], [17], while the larger accuracy of gene networks has been confirmed through functional convergence across groups of affected genes [13]–[16].

### Altered amygdala-cingulate corticolimbic regional gene synchrony in depression

**Figure 3:**
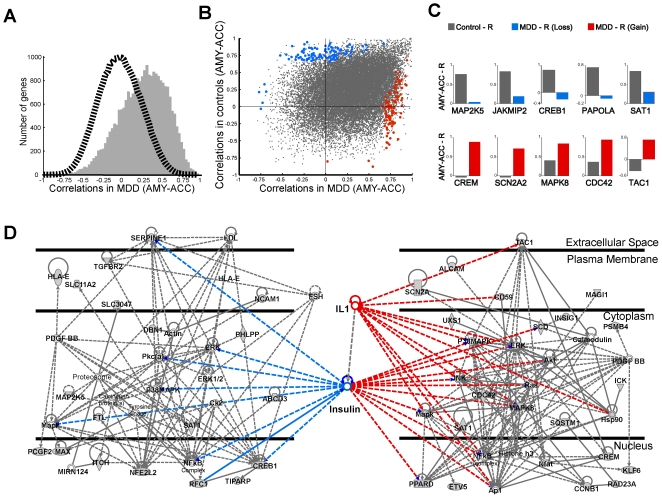
Altered amygdala-cingulate regional gene synchrony in subject with major depression. **A** Global right-shifted histogram of gene synchrony in subjects affected with depression. **B** Comparing gene synchrony between control and depressed subjects confirmed that genes are similarly regulated in both groups (Gray dots) and identified numerous genes with robust decrease (Blue dots: High R in controls, low R in depression) or increase (Red dots: low R in controls, high R in depression) in amygdala-cingulate synchrony. **C** Single gene examples. **D** The top genes and molecule interaction networks built on genes selected with increased (right) or decreased (left) amygdala-cingulate synchrony in depression share similarities in signal transduction components and were linked through insulin, a homeostatic modulator with significant links to both networks (**Table 1**). IL1 was significantly connected to the network build in genes with increased regional synchrony in depression. Grey, depression-affected genes; White, genes or bioactive molecules significantly connected to the network.

We next tested the hypothesis that pathological mechanisms leading to depression may affect the coordination of gene expression patterns between the amygdala and cingulate cortex. Array data from subjects with major depression [10] (n = 14; File S1) displayed a similar amygdala-cingulate gene transcript right-shifted correlation distribution (Figure 3A; median r = 0.32; MDD/Permutated data, p<0.000001). Transcript synchrony was similar in control and depressed subjects (Gray dots in Figure 3B; p<0.000001); hence independently confirming amygdala-cingulate regional gene synchrony in human subjects. Relying on permutation testing procedures to ensure statistical significance of coregulation measures and after controlling the false discovery rate (FDR) (10–30%), as many as 94 gene transcripts displayed robust significant loss of regional synchrony in depression (Blue in Figure 3b–c; from greater to 0.7 to less than 0.2 values), while over 180 displayed significant gain of synchrony in depression (Red in Figure 3B–C; from less than −0.7 to greater than −0.2 values) (File S2).

### Genes with altered transcript synchrony participate in biological networks and signal transduction pathways modulated by hormonal factors previously implicated in depression

To gain insight into biological functions affected by these changes, we overlaid the top 80 genes (∼20% FDR) under both conditions (gain or loss of synchrony) onto the global molecular network of the Ingenuity knowledge database. The top gene networks in both categories displayed similarities, as they included numerous signal transduction and transcription components of the mitogen-activated protein kinase pathway (Table 1) and other genes previously implicated in depression (CREB1 [24], SAT1 [25]). The unbiased inclusion of additional nodes significantly linked to depression-affected genes identified insulin–a recurrently-suggested contributor to neuropsychiatric disorders - as a putative modulator for both networks (Figure 3D; Table 1). The pro-inflammatory cytokine, interleukin 1 (IL1) was identified as a second putative modulator (p<0.0001) for the top network formed by genes with elevated synchrony in depression (Figure 3D; Table 1). Additional biological modulators identified in the top three networks in each category included thyroid hormone, a clinically-useful antidepressant-augmenting agent [26], and beta-estradiol, the major brain estrogen. All associations of the identified modulators with the top gene networks were significant (p<0.05), as assessed by bootstrap resampling. These associations were also selective (p<0.01), as assessed by repeated testing of the Ingenuity database with random gene lists of equivalent or variable sizes of selected genes (60 to 200 genes). Pathways, biological functions and diseases associated with altered gene synchrony are summarized in Table 1. Notably, glucocorticoid receptor signaling was the top canonical pathway associated with three of the top networks, linking stress hormone-related events - a well-characterized causative factor [27] - to the deregulated molecular state in depression.

**Table 1:** Top 3 biological networks formed by genes with gain of loss of amygdala-cingulate gene synchrony.

Net-work	Genes & Molecules in Network	Score	Focus Genes	Canonical pathways	Major function	Disease
**HIGH AMY-ACC gene transcript synchrony in MDD (GAIN OF FUNCTION)**						
1	Akt, **ALCA**M, Ap1, Calmodulin, **CCNB1, CD59, CDC42, CREM**, ERK, **ETV5**, Histone h3, Hsp90, **ICK**, **IL1**, **INSIG1**, **INSULIN**, Jnk, **KLF6, MAGI1**, Mapk, **MAPK8**, Nfat, NFkB (complex), P38 MAPK, PDGF BB, **PPARD, PSMB4, RAD23A**, Ras, **SAT1, SCD, SCN2A, SQSTM1, TAC1, UXS1**	40	20	**Glucocorticoid receptor signaling**; B-cell receptor signaling; PPAR signaling; Xenobiotic metabolism; interleukin signaling	Protein kinase cascade; intracellular signaling	Genetic disorder
2	**AACS, ABCB10, AMACR**, C14ORF106, CABC1, CBLL1, **CCDC106**, CDKL3, CREBL2, **CRYZ**, FAM62A, **FBXO9**, HNF4A, **IER5**, LRRC8C, **MRPL18**, MRPS15, MRPS27, **NDFIP1**, PLA2G12B, **RGNEF**, RNASE4, **RPS18, SCG2**, SH3BGRL2, SREBF1, STK11, TMEM87B, TP53, TPRKB, **TRIM4**, TUBE1, **WDR77**, YWHAZ, ZNF175	27	14		Mitochondrion, intracellular organelle, cellular metabolism, ribosome	Neurolo-gical disorder
3	ALDH1B1, **ATF7IP2**, **BETA-ESTRADIOL**, CNN2, CORO1C, CTNNA2, CTNNB1, DSC3, F2, **GLRB**, GPR137B, GRB2, IFNB1, IFRD2, IKBKE, LAGE3, LOC284230, LRRFIP2, **MAOB**, MYC, **PDHB, PFDN5, PKP4, PUM1, RPL35**, RPL21, SLC11A1, SLC16A5, SP1, **SPTLC1, TAGLN3, TGOLN2, UBAP2L**, VCL, **ZXDB**	24	13		cell-cell junction, cell adhesion, actin-binding	-
**LOW AMY-ACC gene transcript synchrony in MDD (LOSS OF NORMAL CONTROL FUNCTION)**						
1	**ABCD3**, Actin, Calcineurin protein(s), Ck2, **CREB1, DBN1**, ERK, ERK1/2, FSH, **FTL, HLA-E**, **INSULIN**, **ITCH**, LDL, **MAP2K5**, Mapk, **MAX**, MIRN124, **NCAM1, NFE2L2**, NFkB (complex), P38 MAPK, **PCGF2**, PDGF BB, **PHLPP**, Pkc(s), Proteasome, **RFC1, SAT1, SERPINE1, SLC11A2, SLC30A7, TGFBR2, TIPARP**, tyrosine kinase	41	19	**Glucocorticoid receptor signaling**; Xenobiotic metabolism, NRF2-mediated oxidative stress response; B-Cell receptor signaling; PPAR signaling	Protein kinase; intracellular signaling; Phosphoprotein	-
2	**ABHD5**, Akt, **BETA-ESTRADIOL**, **CTNNA1**, CTNNB1, **DDX21, GPNMB, HNRPDL, HR**, IL4, IL13_GC_, **JAKMIP2**, MAP3K1, MIRNLET7G, MYC, **PDK1, PNN**, RELA, **SAR1B, SLC26A2**, SMAD3, **SMCR7L, SOCS4**, SPRR2B, SPRR2G, **STAG2, TFCP2**, TGFB1, **THYROID HORMONE**, **TNS1, TUBB6**, YWHAG, ZFP161, **ZMYM2, ZNF337**	40	19	**Glucocorticoid receptor signaling**	Regulation of cellular process: transcription, metabolism, cell growth	-
3	ADSS, APEH, **ARMC6**, C11ORF58, CRIPT, DLG4, **EML1**, **GGCX**, GRID2, GTPBP3, GYS2, HLA-B, HNF4A, HSPC152, **LILRB3, LRRC1**, LRRC40, MPP1, **NLGN4X**, OGDH, **PAPOLA, PPP1R10**, PPP1R11, PPP1R3D, PPP2R5B, PTPRG, **RIOK2**, SEC11A, **SEPT2, SGCE**, SLC25A1, SRP68, **STYXL1**, TPP2, **WDR42A**	25	13		Protein phosphatase activity; Cytoskeleton	-

In double-underline are biological modulators significantly connected to the network over a range of FDR's and unlikely to be selected at random (p<0.01). Depression-affected genes are in bold. Other included genes/molecules displayed significant interactions with Depression-affected genes in network. “Canonical pathways” contain genes linked to ≥25% of nodes in networks. AMY, amygdala; ACC, anterior cingulate cortex; MDD, major depressive disorder.

## Discussion

Our findings demonstrate that regional gene synchrony, as measured by gene-wise correlated transcript levels across brain regions within individuals, is a major component of gene expression patterns in the human brain (Figure 1). These patterns were not explained by genetic, age or microarray effects, and appeared driven by correlation within subjects, as scrambling the data across subjects abolished them (Figure 1E–F, Figure 2). Thus we speculate that regional gene synchrony may partly reflect an integrated molecular output of function-, and dysfunction-dependent, regulation of brain areas.

While data from the amygdala/cingulate cortex and from the two prefrontal cortex areas supports the contention that gene coordination may reflect an overall, or network-specific, concerted brain region function, our results are independent of these larger hypotheses, as we only considered here the functional significance of those genes which show significant depression-related alteration in gene synchrony between two regions known to be functionally affected by depression. Accordingly, by bootstrapping correlations and controlling the FDR, we identified a robust and conservative collection of genes that displayed significant gains or losses of amygdala-cingulate gene transcript coordination in subjects with depression (Figure 3) (which is distinct from mean absolute expression level changes; See Comments section). These gene sets implicated shifts in intracellular signaling, metabolism and cell growth/structure, and suggested the implication of several biological modulators previously associated with depression (Table 1).

Notably, changes in amygdala-cingulate gene synchrony suggest a combined dysregulated function for several hormone-type modulators (Figure 3D; Table 1), which together summarize several key hypotheses for pathophysiological mechanisms in depression. As postmortem studies preclude investigating short-term events, we propose that the present findings may correspond to a stable, chronic and adaptive *de novo*, although pathological, state. The integrative nature of this deregulated state departs from reductionist approaches and has critical implications for our understanding and modeling of pathological mechanisms of depression.

### What may underlie regional gene synchrony

As coregulated gene expression reflect the influences of genetic, biochemical and environmental factors [13], [28], we speculate that the observed gene synchrony across regions may reflect a molecular balance of local brain systems that is achieved over time (days-months) through the coordinated function and continuous feedback of interacting brain regions. For instance, starting at the cellular level, the rate of neuronal firing is determined by the molecular composition of local neuronal circuits. The cumulative electric signals of single neurons with neuronal ensembles oscillate on various timeframes, supporting regional brain function and underlying correlated functions across regions. In turn, the translation and integration of neuronal activity by intracellular signaling cascades is influenced by coordinated activities across functionally-related brain regions. Additionally, broad and long-acting modulators (hormones-type factors) modulate transcriptional programs (through nuclear receptors, for instance) and interact/modify this conversion of neuronal activity into cellular changes over time and across areas. According to this model, the disturbances in biological rhythms observed in depression (circadian, hormonal cycles) and the known role of environmental exposure (stress, disease) in precipitating disease episodes, will influence the degree of cellular exposure to hormone-type modulators, and may potentially result in an altered, yet stable, molecular balance. This suggested mechanism resembles a “decanalization” process that has been proposed for complex disorders, where chronic shifts in various regulating factors converge to induce and maintain a departure from the biologically-optimized healthy organism into a distinct, stable and maladaptive pathological state [29].

### A combined hormone-mediated disease pathology in major depression

Consistent with the above-proposed model, the unbiased analysis of biological modulators associated with networks formed by genes with altered amygdala-cingulate coregulation, identified factors previously implicated in depression or in its treatment (insulin, beta-estradiol, thyroid hormone, IL1 and glucocorticoids; Table 1), although notably, none of them would be sufficient to reasonably explain the presence of the illness in heterogeneous clinical cohorts [1], [24]. ***(1)*** Insulin shared potential control over genes forming the most robust networks under conditions of gain and loss of synchrony (Figure 3), suggesting that deregulation of this homeostatic modulator may participate in mediating pathological changes in depression. Insulin has been suggested as a potential mediator of metabolic changes in neuropsychiatric disorders [30], whereas insulin-resistance is more frequent in subjects with familial depression [31]. ***(2)*** Thyroid hormone influences brain physiology through regulating basal metabolism and neuronal maturation. Low thyroid function is associated with increased incidence of depression, while thyroid adjuvant therapy augments antidepressant therapy, potentially through deactivation of limbic regions [26]. ***(3)*** Although associated with mood changes in female subjects, the relevance of altered estradiol function to this male group is underscored by local aromatase–mediated conversion of testosterone to estradiol, including in the amygdala, where it modulates anxiety and depressive-like behaviors [32]. ***(4)*** IL1 is a potent pro-inflammatory cytokine, which mediates aspects of the “sickness behavior”, a syndrome sharing similarities with major depression [33]. Interestingly, IL1 was associated here with increased regional gene synchrony in depression, suggesting a gain-of-function mechanism consistent with IL1 recruitment and role. ***(5)*** Finally, as potential core inducing-factors of the illness [27], glucocorticoids (and stress) are known to modulate the functions of all other identified factors, resulting among others in altered blood brain barrier function, decreased glucose uptake, immune activation, and disrupted sex hormone cycling or release [26], [33], [34]. Circulating interleukin and other cytokines also affect insulin function [33], together suggesting that a complex interplay of disrupted hormone-mediated regulations of organs and cell ensembles may occur in depression.

Hence, as contributing roles in depression are separately consistent for all identified factors, it is conceivable to envision a model where sustained environmental and lifestyle changes induce chronic adaptive changes in several systems (insulin, sex-hormones and thyroid-related functions), which now interacts with individual genetic make-up or additional environmental disturbances (stress or infection). Thus this model connects and potentially synergizes distinct and previously-proposed pathophysiological mechanisms for depression. Accordingly, pathways to a depressive state are not likely to be explained by any single factor, but may reflect the disruption of several hormone-type factors acting on different timeframes (cyclic, constant, phasic or induced). Here our results suggest that in depression these factors may converge on intracellular pathways (e.g., MAPK pathway), mitochondria and energy metabolism, and on other neurochemical pathways, such as SAT1 and polyamines [25], resulting in altered function and cell structure (growth, adhesion) within the amygdala-cingulate network (Table 1). In conclusion, we propose that the identified departures in corticolimbic regional gene synchrony represent an integrated gene/molecular signature of a *de novo* maladaptive and pathological state in subjects with major depression.

### Limitations and comments

- The present findings demonstrate that regional gene coordination represents a biological feature of the human brain across related areas, and that alterations in this phenomenon are useful for measuring integrated multi-scale effects in complex disorders such as depression. Our results provide the basis for further mapping of gene coordination structure onto specific functional brain networks. Indeed, the extent to which gene coordination follow the boundaries of known anatomical/functional networks is still to be determined. For instance, the amygdala is an anatomical and functional hub [35] and we may expect positive large-scale gene synchrony with other, but not all areas.

- Similarly, the question of a control brain region in depression is often discussed but not clarified. Indeed, it is not know whether the primary pathology of the illness is region-specific or widespread and data has been provided for both cases [10], [36]. So in short, there is no consensus for a “control” brain region in depression. An additional practical limitation is that no similar datasets are currently available to define the limits of the effect of MDD on regional gene coordination. Finally, critical to our findings, our results do not depend on other regions being affected or not, but instead provide information on gene coordination in areas of a network that is affected in depression. Whether other brain regions are affected is an important scientific question, but for which the answer is complementary rather than necessary for the current study.

- With regards to validating gene coexpression, it is becoming increasingly clear that there are different types of independent validation of array data for: *1)* differential expression level, *2)* coregulation and *3)* functional implications, which in turn require different analytical approaches. Coregulation relies on changes of small magnitude across samples that are difficult to replicate by qPCR. Our results provide a technical reason for the usual absence of such confirmatory approaches in coregulation studies, which is that the variability of qPCR measurement is higher than the one observed in array data. Such information had to our knowledge not yet been provided in the rapidly growing field of coregulation studies. Instead, coregulation methods rely on assessing the probability of confirming the observed effects in other array datasets and on the probability of results belonging to common biological pathways. Here, we provide very robust statistical findings for these two types of validation, using bootstrap and other resampling statistical approaches. An implication of these observations is that it may not be wise to rely on single key genes as modulators of the observed effects, as the statistical reliability of any individual genes is moderate, compared to the robust statistical significance of coregulated or functionally-related gene groups. Here we relied on a process of convergent confirmation of mediators of depression, across groups of genes through the well-validated Ingenuity's literature-based database.

- The mostly positive coordinated patterns may be surprising, based on known biological interactions between areas and cell types. Studies in regions with well-characterized neurotransmitter structures, such as raphe/cortex or ventral tegmental area/nucleus accumbens may help resolve this question. Notably, the proposed assay (regional gene synchrony) does not identify a single area of origin of disease-related changes (an intrinsic limitation of the approach), but rather suggests changes in factors supporting synchronization of gene function across brain areas in depression.

- Other factors are likely engaged in the illness, for which the size and composition of the cohorts did not allow us to identify. For instance, early developmental events and indirect modulation (e.g. through monoamine regulation) may be more challenging to identify and are not necessarily well characterized in currently available functional gene networks. Finally, although we ruled out the contribution of several factors (genetic variants, tissue-specific programs, age) we only speculate that function-dependent regulation may be at play in supporting the depression-related correlation shifts. Hence, it will be of critical interest to assess whether correlated patterns return to control states in remitted subjects and if such patterns are measurable in rodent models of the illness. Finally, it is notable that robust coordinated patterns were observed in a relatively small and heterogeneous cohort of subjects. Indeed, it is likely that demographic and clinical parameters, such as sex, race, lifetime stress exposure and antidepressant exposure for instance, will influence regional gene synchrony, although much larger cohorts and multi-region gene arrays datasets will be necessary to investigate the full extent of these effects. Despite all of these potentially confounding influences, gene coordination remains a strong influence on patterns of gene expression across areas, and for which alterations in depressed subjects correspond to known and suspected abnormalities in the illness.

## Materials and Methods

### Cohort description and array parameters

Human cohort 1 (amygdala and cingulate) includes samples from 28 white male subjects: 14 control subjects and 14 subjects with familial major depression. Subject description, array sampling and parameters were previously described [10]. In brief, brain samples were obtained at the Allegheny County Medical Examiner's Office (Pittsburgh) after written consent from next-of-kin. Consensus DSM-IV diagnoses were made by an independent committee of experienced clinical research scientists, utilizing information from clinical records, toxicology exam and a standardized psychological autopsy. Depressed and normal comparison subjects were matched for age, sex and race (File S1). Amygdala samples were dissected from frozen coronal blocks ∼2–3 cm caudal to the temporal pole and were enriched in lateral, basolateral and basomedian nuclei. Cingulate samples were harvested from coronal sections in subgenual cingulate and contained all six cortical layers. All procedures were approved by the University of Pittsburgh's Institutional Review Board and by the Committee for Oversight of Research Involving the Dead. The second human cohort (BA9 and 47) includes 19 control subjects. Subjects description, array parameters and data are available in [37]. Details about arrays processing and parameters are summarized in the supplemental files.

### Statistical methods

Gene-wise bootstrapped Pearson correlation r-values were used to ensure an accurate estimation of the “real” underlying distribution and to avoid spurious findings due to outlying data. Because gene coordination relies on multiple samples to form a single measure, we used the percentile bootstrap method to ensure that the shifts were robust and significant (p<0.05) and then applied the Benjamini-Hochberg FDR [38]. To increase the power of the analysis, we considered alterations of coordination in genes with high correlations in at least one condition, which are indicative of inter-regional communication [18].

While methods exist to optimize cutoff values in coexpression networks [28], there are no analogous mathematical methods for coordinated expression for the same genes. Therefore we used a 0.7 r-value cutoff (resulting in = 3244 probesets with cross-area links) that was indicated as an optimal balance of false positives and false negatives for within-area amygdala and cingulate networks [28], and results did not significantly vary for alternative cutoffs (+/−0.1) (not shown). To generate p-values that quantify the depression-related shift in gene coordination, we used the percentile bootstrap method. These p-values for shifts in correlation were estimated using 20,000 bootstrap resamples of the raw data, at which point p-values were stable.

### Methods for eliminating age correlation in microarray data

While baseline comparison of age (Figure 2B) did not show any influence of age-correlated genes on the amygdala-cingulate expression correlations (r = −0.01), to avoid ambiguity in the source of gene coordination we detrended any linear relationship with age in both amygdala and cingulate data (eliminating the possible influence of any large magnitude y-values shown in figure 2B on gene coordination). Because the final correlations used to assess gene coordination were bootstrapped, to detrend the data we removed any linear relationship with age in each of the 20,000 bootstrap instances and used these detrended resamples to generate a histogram of amygdala-cingulate expression profiles as before (see Figure 2A).

### Biological pathway, gene network and modulator analyses

Selected genes were overlaid on the global molecular network developed from information contained in the Ingenuity Pathway knowledge base (www.ingenuity.com). This network is composed of ∼2 million literature-based biological links between genes and bioactive molecules, and sub-networks are built on genes of interest based on their connectivity within this global network. Gene networks were limited to 35 nodes. The score for a network takes into account the relative numbers of network eligible molecules, of molecules analyzed and the total number of molecules in Ingenuity's knowledge base. These scores are based on the hypergeometric distribution and represent the negative log of the right-tailed Fisher's Exact Test p-value. Disease links are based on literature-based association with illness. The major functions of gene clusters were determined by DAVID functional clustering (http://david.abcc.ncifcrf.gov).

To assess whether the association of the identified biological modulators with the top networks was specific (p<0.01), we resampled the Ingenuity database 100 times of with random gene lists of equivalent sizes. The process was repeated with variable sizes of selected genes (60 to 200 genes). Finally, the probability of finding the identified modulators in the top gene networks was assessed bootstrap resampling.

## Supporting Information

File S1 Cohort descriptions and array parameters; AMY-ACC probeset correlations.
(0.12 MB PDF)

File S2 Complete per-probeset amygdala-anterior cingulate cortex coordination levels and p-values.
(0.75 MB XLS)
