# Supplementary material for: Altered Gene Synchrony Suggests a Combined Hormone-Mediated Dysregulated State in Major Depression
Source: PLoS One. 2010 Apr 1;5(4):e9970. doi: 10.1371/journal.pone.0009970 (PMC2848620; doi:10.1371/journal.pone.0009970)
Supplement: File S1 — Cohort descriptions and array parameters; AMY-ACC probeset correlations. (0.12 MB PDF) [file pone.0009970.s001.pdf]

Supplemental information

Altered Gene Synchrony Suggests a Combined Hormone-Mediated Dysregulated State in Major Depression

Chris Gaiteri[1,2], Jean-Philippe Guilloux[1,3], David A. Lewis[1,2] & Etienne Sibille[1,2]

[1] Department of Psychiatry, [2] Center for Neuroscience, University of Pittsburgh, Pittsburgh, Pennsylvania, USA, [3] Université Paris-Sud EA 3544, Faculté de Pharmacie, Châtenay-Malabry cedex F-92296, France.

* Corresponding author. Department of Psychiatry, University of Pittsburgh, 3811 O'Hara street, BST W1643, Pittsburgh, PA 15213, USA. Tel.; 1 412 624 0804; Fax.: 412-624-9910. E-mail: sibilleel@upmc.edu.

Table of contents *Pages*

1. Cohort description and array parameters (including Table S1). *2-4*

2. Table S2. Complete amygdala-cingulate probeset correlations. *5*

3. References *6*

---

1. Cohort description and array parameters.

1.1. Human cohort 1 (amygdala and cingulate) was described in details in [1,2]. A brief summary is provided here. In brief, brain samples were obtained during autopsies conducted at the Allegheny County Medical Examiner's Office (Pittsburgh) after consent from next-of-kin. Control subjects did not have an Axis I psychiatric disorder, were antidepressant drug-free and died from natural or accidental causes other than suicide. Subjects with advanced disease stages (i.e., cancer, neurodegenerative disorders) were excluded. Control subjects were paired to each case as closely as possible on age and freezer storage time. (Table S1). For all subjects, consensus DSM-IV diagnoses of MDD were made by an independent committee of experienced clinical research scientists at a case conference utilizing information obtained from clinical records,

toxicology exam and a standardized psychological autopsy [3]. MDD subjects with co-morbid psychiatric disorders were excluded. Antidepressant drug exposure was assessed by clinical data from structured interviews, review of records and toxicology studies. All cases and controls were white Caucasian and were selected for rapid modes of death and short agonal phases, to limit the influence of agonal factors on RNA quality and pH [4]. All selected brains were analyzed for adequate brain pH (>6.4) and RNA integrity by optical density (OD≥ 1.6) and Agilent bioanalyzer analysis (Agilent Technologies, Palo Alto, CA; RIN expert scoring system ≥ 7) as previously described [5]. All procedures were approved by the Institutional Review Board and Committee for Oversight of Research Involving the Dead.

	Major depression (MDD) group						Control group					
Pair	Case	Age	PMI	pH	Storage	RIN	Case	Age	PMI	pH	Storage	RIN
1	505	57	12.8	7.1	125	8.7	685	57	14.0	6.6	98	8.0
2	513	25	13.1	6.9	123	7.0	789	23	20.0	7.0	80	7.4
3	598	69	5.9	7.3	111	8.8	795	69	12.0	6.6	102	8.2
4	600	64	9.9	6.7	110	7.1	615	62	7.2	6.4	108	7.8
7	698	59	13.0	6.8	96	9.0	713	58	37.5	7.0	94	8.4
8	783	63	11.5	6.5	82	8.8	736	55	15.5	6.9	88	8.3
9	863	52	28.3	7.2	65	8.4	1086	51	24.2	6.8	27	8.1
10	868	47	10.5	6.8	64	8.1	857	48	16.6	6.7	66	8.9
11	926	57	19.0	7.0	20	7.3	1122	56	15.4	6.7	49	7.9
12	1001	54	7.3	6.6	67	7.6	852	54	8.0	6.8	36	9.1
13	1049	48	5.4	6.6	30	8.4	1067	49	6.0	6.6	77	8.2
14	809	50	20.0	6.9	77	8.5	1031	54	23.1	6.8	35	8.2
15	1060	30	11.1	6.6	97	8.3	604	39	19.3	7.1	108	8.6
16	943	56	15.4	6.6	26	8.2	1047	43	12.0	6.9	32	9.0
Avg		52.2	13.1	6.8	77.9	8.2		51.3	16.5	6.8	71.2	8.3
Stdev		12.2	6.1	0.2	35.0	0.7		11.1	8.2	0.2	30.3	0.5

Table S1. MDD and control cohorts. *Age (years); PMI, postmortem interval (Hours); Storage, freezer storage at -80◦C (months); RIN, RNA integrity number; Average values for age, PMI, pH, storage time and RNA quality were not different between MDD and control groups (all p>0.05).*

Microarray processing and parameters. *(See details in [2])* Rostral AMY samples enriched in lateral, basolateral and basomedian nuclei were utilized. ACC samples containing all six cortical layers were harvested in rostral subgenual ACC. Total RNA were extracted from frozen samples stored in TRIZOL (Invitrogen, Carlsbad, CA) and processed onto Human Genome U133Plus-2.0 arrays (Affymetrix, Santa Clara, CA) according to manufacturer's protocol. Hybridization and arrays quality control, probeset signals (i.e., transcript level intensities) were extracted with the Affymetrix Microarray GCOS software. For statistical analysis, Log_2-transformed probeset signal intensities

were extracted and normalized with the GC- Robust Multi-array Average (GC-RMA) algorithm [6]. Individual scans were visually inspected for the presence of manufacturing defaults and hybridization artifacts. Quality control parameters were as follows: noise (RawQ, ACC, 1.47±0.34; AMY, 1.47±0.34), background (ACC, 44±8; AMY, 43±5), scale factor (ACC, 2.62±1.32; AMY, 4.41±0.97), 3'/5' Actin ratio (ACC, 2.97±0.98; AMY, 2.98±0.93) and 3'/5' GAPDH ratio (ACC, 1.23±0.217; AMY, 1.45±0.28). Consistent number of genes were detected as present across arrays (ACC, 46.8±2.2%; AMY, 48.2±3.3%), and consistent detection of BioB/C hybridization spiked controls. See [2] for details.

1.2. **Human cohort 2 (BA9 and 47)** includes 19 control subjects. Subjects description, array parameters and data are available in the three publications listed below. Similar array processing and parameters as in cohort 1 were applied.

- Galfalvy,H.C., Erraji-Benchekroun,L., Smyrniotopoulos,P., Pavlidis,P., Ellis,S.P., Mann,J.J., Sibille,E., and Arango,V. (2003). Sex genes for genomic analysis in human brain: internal controls for comparison of probe level data extraction. BMC Bioinformatics 4, 37.

- Sibille,E., Arango,V., Galfalvy,H.C., Pavlidis,P., Erraji-BenChekroun,L., Ellis,S.P., and Mann,J.J. (2004). Gene expression profiling of depression and suicide in human prefrontal cortex. Neuropsychopharmacology 29, 351-361.

- Erraji-BenChekroun,L., Underwood,M.D., Arango,V., Galfalvy,H.C., Pavlidis,P., Smyrnioto-poulos,P., Mann,J.J., and Sibille,E. (2005). Molecular aging in human prefrontal cortex is selective and continuous throughout adult life. Biological Psychiatry 57, 549-558.

2. Table S2. Complete AMY-ACC probeset correlations.

Correlation and p-values were computed using the percentile bootstrap (see statistical methods). Genes with p-values that survive a false discovery rate (FDR) cutoff of less than or equal to 30% are noted in 5% increments. Only genes with correlations greater than 0.7 or less than -0.7 in one condition were selected for differential analysis.

A sample of the table is displayed below. The full table is 37 pages long is available as a separate downloadable Excel file.

probeset	Gene symbol	Gene Title	Ctrl R	MDD R	MDD-Ctrl (ΔR)	Low in MDD p-value	sig. at FDR of .	High in MDD p-value	sig. at FDR of .
1053_at	RFC2	replication factor C (activator 1) 2, 40kDa	0.435	0.892	0.456	0.9415		0.0585	
1552274_at	PXK	PX domain containing serine/threonine kinase	0.684	0.442	-0.241	0.0808		0.9192	
1552283_s_at	ZDHHC11	zinc finger, DHHC-type containing 11	0.724	0.798	0.074	0.7316		0.2684	
1552310_at	C15ORF40	chromosome 15 open reading frame 40	0.409	0.709	0.300	0.81605		0.18395	
1552426_a_at	TM2D3	TM2 domain containing 3	0.303	0.700	0.396	0.9626		0.0374	
1552621_at	POLR2J2	polymerase (RNA) II (DNA directed) polypeptide J2	0.722	0.825	0.103	0.7588		0.2412	
1552626_a_at	TMEM163	transmembrane protein 163	-0.184	0.733	0.917	0.996		0.004	0.25
1552701_a_at	CARD16	caspase recruitment domain family, member 16	0.705	0.113	-0.592	0.01815		0.98185	
1552722_at	ARPP-21	cyclic AMP-regulated phosphoprotein, 21 kD	0.703	0.389	-0.315	0.1135		0.8865	
1552931_a_at	PDE8A	phosphodiesterase 8A	0.699	0.469	-0.230	0.2089		0.7911	
1553037_a_at	SYN2	synapsin II	0.788	0.564	-0.224	0.22755		0.77245	
1553108_at	C5ORF24	chromosome 5 open reading frame 24	0.749	0.089	-0.661	0.0237		0.9763	
1553118_at	THEM4	thioesterase superfamily member 4	0.715	0.549	-0.166	0.24055		0.75945	
1553133_at	C9ORF72	chromosome 9 open reading frame 72	0.916	0.501	-0.415	0.2216		0.7784	
1553193_at	ZNF441	zinc finger protein 441	0.765	0.476	-0.289	0.19215		0.80785	
1553271_at	DIP2B	DIP2 disco-interacting protein 2 homolog B (Drosophila)	0.396	0.707	0.311	0.90705		0.09295	
1553551_s_at	ND2	NADH dehydrogenase, subunit 2 (complex I)	0.696	-0.218	-0.914	0.0037	0.2	0.9963	
1553677_a_at	TIPRL	TIP41, TOR signaling pathway regulator-like (S. cerevisiae)	0.671	0.747	0.076	0.60555		0.39445	
1553704_x_at	ZNF791	zinc finger protein 791	0.699	0.570	-0.129	0.2681		0.7319	
1553796_at	FLJ30594	hypothetical locus FLJ30594	0.730	-0.043	-0.774	0.00125	0.2	0.99875	
1553864_at	DKFZP761H2121	hypothetical protein DKFZp761H2121	0.272	0.771	0.499	0.988		0.012	0.3
1553971_a_at	TCAG7.1177	opposite strand transcription unit to STAG3	0.339	0.800	0.461	0.92145		0.07855	
1554004_a_at	RGNEF	Rho-guanine nucleotide exchange factor	0.070	0.887	0.817	0.99725		0.00275	0.25
1554018_at	GPNMB	glycoprotein (transmembrane) nmb	0.806	0.686	-0.120	0.2927		0.7073	
1554093_a_at	SNAPC5	small nuclear RNA activating complex, polypeptide 5, 19kDa	0.557	0.734	0.177	0.6309		0.3691	
1554181_at	SNX32	sorting nexin 32	0.645	0.757	0.112	0.68115		0.31885	
1554287_at	TRIM4	tripartite motif-containing 4	-0.169	0.804	0.972	0.99695		0.00305	0.25
1554306_at	ITPKB	inositol 1,4,5-trisphosphate 3-kinase B	0.885	0.237	-0.647	0.1234		0.8766	
1554351_a_at	TIPRL	TIP41, TOR signaling pathway regulator-like (S. cerevisiae)	0.677	0.698	0.021	0.5454		0.4546	
1554452_a_at	HIG2	hypoxia-inducible protein 2	0.996	0.263	-0.733	0.074		0.926	
1554482_a_at	SAR1B	SAR1 homolog B (S. cerevisiae)	0.721	-0.104	-0.825	0.0044	0.2	0.9956	
1554509_a_at	C10ORF97	chromosome 10 open reading frame 97	0.364	0.776	0.412	0.89985		0.10015	
1554593_s_at	SLC1A6	solute carrier family 1 (high affinity aspartate/glutamate trans	0.204	0.692	0.488	0.926		0.074	
1554643_at	RGS11	regulator of G-protein signaling 11	0.184	0.731	0.547	0.81055		0.18945	
1554689_a_at	NLGN4X	neuroligin 4, X-linked	0.829	0.244	-0.585	0.0041	0.2	0.9959	
1554706_at	OR2L13	olfactory receptor, family 2, subfamily L, member 13	0.627	0.824	0.197	0.779		0.221	
1554747_a_at	2-Sep	septin 2	0.845	0.179	-0.666	0.0007	0.15	0.9993	
1554789_a_at	PDE8B	phosphodiesterase 8B	0.682	0.645	-0.037	0.444		0.556	
1554801_at	C5ORF40	chromosome 5 open reading frame 40	0.133	0.806	0.672	0.99395		0.00605	0.25
1554810_at	PLA2G4C(include	phospholipase A2, group IVC (cytosolic, calcium-independe	0.058	0.840	0.781	0.9114		0.0886	
1555123_at	ST6GAL2	ST6 beta-galactosamide alpha-2,6-sialyltranferase 2	0.528	0.680	0.152	0.82325		0.17675	
1555241_at	C8ORF59	chromosome 8 open reading frame 59	0.781	0.603	-0.179	0.2401		0.7599	
1555311_at	RAB22A	RAB22A, member RAS oncogene family	0.092	0.796	0.704	0.92255		0.07745	
1555318_at	HIF3A	hypoxia inducible factor 3, alpha subunit	0.852	0.364	-0.488	0.0333		0.9667	
1555363_s_at	MGC39821	hypothetical protein MGC39821	0.718	0.051	-0.667	0.06545		0.93455	
1555618_s_at	SAE1	SUMO1 activating enzyme subunit 1	0.681	0.539	-0.142	0.30315		0.69685	
1555653_at	HNRNPA3	heterogeneous nuclear ribonucleoprotein A3	0.794	0.427	-0.367	0.07		0.93	
1555764_s_at	TIMM10	translocase of inner mitochondrial membrane 10 homolog (y	0.408	0.742	0.334	0.8313		0.1687	
1555812_a_at	ARHGDIB	Rho GDP dissociation inhibitor (GDI) beta	0.697	0.249	-0.447	0.11165		0.88835	
1555832_s_at	KLF6	Kruppel-like factor 6	0.752	0.730	-0.022	0.43015		0.56985	
1555847_a_at	LOC284454	hypothetical protein LOC284454	0.238	0.803	0.565	0.70435		0.29565	
1555881_s_at	LZTS2	leucine zipper, putative tumor suppressor 2	0.206	0.723	0.517	0.9575		0.0425	
1555892_s_at	LOC253039	hypothetical LOC253039	0.886	0.798	-0.089	0.1896		0.8104	
[illegible]	[illegible]	[illegible]	[illegible]	[illegible]	[illegible]	[illegible]		[illegible]	

3. Reference List

1. Sibille E, Arango V, Joeyen-Waldorf J, Wang Y, Leman S *et al* (2008) Large-scale estimates of cellular origins of mRNAs: Enhancing the yield of transcriptome analyses. J Neurosci Methods 1672: 198-206.

2. Sibille E, Wang Y, Joeyen-Waldorf J, Gaiteri C, Surget A *et al* (2009) A molecular signature of depression in the amygdala. Am J Psychiatry 1669: 1011-1024.

3. Glantz LA, Lewis DA (1997) Reduction of synaptophysin immunoreactivity in the prefrontal cortex of subjects with schizophrenia. Regional and diagnostic specificity. Arch Gen Psychiatry 547: 660-669.

4. Tomita H, Vawter MP, Walsh DM, Evans SJ, Choudary PV *et al* (2004) Effect of agonal and postmortem factors on gene expression profile: quality control in microarray analyses of postmortem human brain. Biol Psychiatry 554: 346-352.

5. Eggan SM, Hashimoto T, Lewis DA (2008) Reduced cortical cannabinoid 1 receptor messenger RNA and protein expression in schizophrenia. Arch Gen Psychiatry 657: 772-784.

6. Irizarry RA, Bolstad BM, Collin F, Cope LM, Hobbs B *et al* (2003) Summaries of Affymetrix GeneChip probe level data. Nucleic Acids Res 314: e15.
